# *Achyranthes bidentata* polysaccharide can safely prevent NSCLC metastasis via targeting EGFR and EMT

**DOI:** 10.1038/s41392-020-00289-2

**Published:** 2020-08-31

**Authors:** Chunlian Zhong, Jingyi Yang, Yusheng Lu, Huanzhang Xie, Shengyi Zhai, Chen Zhang, Zhiying Luo, Xuanchen Chen, Xuanmo Fang, Lee Jia

**Affiliations:** 1 Institute of Oceanography, Minjiang University, Fuzhou, Fujian 350108 China; 2 Cancer Metastasis Alert and Prevention Center, College of Chemistry, Fujian Provincial Key Laboratory of Cancer Metastasis Chemoprevention and Chemotherapy, Fuzhou University, Fuzhou, Fujian 350116 China; 3 Fujian Provincial People’s Hospital Affiliated to Fujian University of Traditional Chinese Medicine, Fuzhou, 350004 China

**Keywords:** Metastasis, Lung cancer

**Dear Editor**,

Cancer metastasis, dissemination of cancer cells from primary tumors to distant sites, attributes to 90% cancer-related death.^1^ Although the targeted therapies, bio-nanomaterial-based target delivery, and the consequent adoption of the so-called personalized medicine have obtained notable achievements in some cancers, significant problems still exist with these approaches.^2^ There are hardly any cancer therapeutic agents that can interfere with metastasized cancer. In recent times, we proposed a novel concept, namely cancer metastasis chemoprevention, which is different from the existing cancer chemotherapy and cancer chemoprevention. Cancer metastasis chemoprevention aims at safely preventing circulating tumor cells (CTCs) and related molecules from gemmating into the metastatic tissues. The strategy does not intend to kill cancer cells as cancer chemotherapy does, nor to aimlessly prevent cancers from carcinogenesis as cancer chemoprevention does. Rather, cancer metastasis chemoprevention interrupts CTCs from adhesion onto the microvascular endothelium of the metastatic tissues. Activation/adhesion of CTCs to the vascular endothelium is a crucial starting point of the metastatic cascade and epithelial-to-mesenchymal transition (EMT) is closely associated with malignancy of tumor, and if we can control the starting point (activation/adhesion) and the EMT progression, we may prevent cancer metastasis. Based on our previous studies,^3^ the biological process of CTC adhesion to the vascular endothelium is similar to embryo implantation into the endometrium. Indeed, abortifacients mifepristone (RU486), metapristone, and ginsenoside R0 isolated from *Achyranthes bidentata* Blume (traditional Chinese medicine, TCM) could inhibit CTC adhesion to vascular endothelium and tumor metastases. Here we show that bioactive polysaccharide isolated from the roots of *A. bidentata* (ABP) and used as a single agent, can safely and effectively prevent cancer metastasis in vitro and in vivo via targeting epidermal growth factor receptor (EGFR) and blocking EMT process.

First, we explored whether ABP was safe enough to be a metastasis chemoprevention agent. ABP showed insignificant cytotoxicity on A549 and PC-9 cancer cells and no obvious inhibition of Human pulmonary microvascular endothelial cells (HPMECs) viability (Supplementary Fig. S1a, b). These results demonstrated that ABP at concentrations up to 100 μg/mL did not affect cell viability. To explore whether ABP has an inhibitory effect on cell adhesion, migration, and invasion, we incubated A549 and PC-9 cells with HPMECs or cell matrix at different concentrations of ABP. ABP produced a concentration-dependent inhibition on adhesion of A549 and PC-9 cells to HPMECs (*P* < 0.05) (Fig. 1a and Supplementary Fig. S2a) and extracellular matrix (*P* < 0.05) (Supplementary Fig. S2b). The cell scratch assay and transwell invasion assays showed that the migration (Fig. 1b and Supplementary Fig. S2c) and invasion (Fig. 1c and Supplementary Fig. S2d) ability of A549 and PC-9 cells treated with ABP was much lower than that of control group (*P* < 0.05). These cell function assays demonstrated that ABP at non-cytotoxic concentrations significantly inhibited adhesion, migration, and invasion of non-small-cell lung cancer (NSCLC) cells in vitro.

**Fig. 1:**
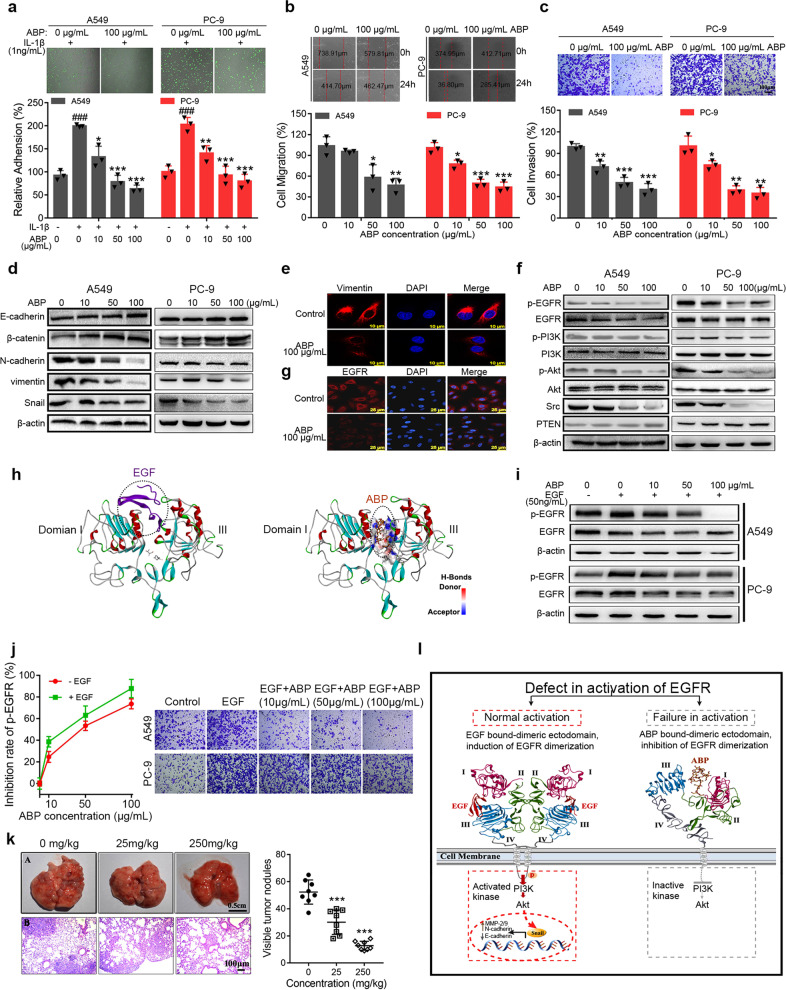
ABP inhibits cell adhesion, migration, and invasion through targeting EGFR. **a** Effect of ABP on adhesion of A549 and PC-9 cells to HPMECs. Upper panel: representative fluorescence microscopic images showed that ABP inhibited the adhesion of rhodamine-123 labeled A549 and PC-9 cells to the HPMECs monolayers stimulated by IL-1β (1 ng/mL). Lower panel: quantitative analyses of the inhibition of ABP on the adhesion of A549 and PC-9 cells to HPMECs. ^###^*P* < 0.001 compared to the control group, *P < 0.05, **P < 0.01, ***P < 0.001 compared to IL-1β-treated group. **b**, **c** Effect of ABP on migration (**b**) and invasion (**c**) of A549 and PC-9 cells. Upper panel: representative micrographs of A549 and PC-9 cells. Lower panel: quantitative analyses of the effect of ABP on cell migration or invasion. The data represented mean ± SD, *n* = 3; *P < 0.05, **P < 0.01, ***P < 0.001 compared to the control group. **d** Western blot analyses of E-cadherin, β-catenin, N-cadherin, vimentin, and Snail protein expressions in A549 and PC-9 cells treated with ABP for 24 h. **e** Double immunofluorescence staining with DAPI and anti-vimentin antibody was carried out for A549 cells treated with ABP for 24 h. **f** Western blot analyses of EGFR, p-EGFR, PI3K, p-PI3K, Akt, p-Akt, Src, and PTEN expression levels in A549 and PC-9 cells treated with ABP for 24 h. **g** Double immunofluorescence staining with DAPI and anti-EGFR antibody was carried out for A549 cells treated with ABP for 24 h. **h** Comparison of computational predicted binding site of ABP and EGF with EGFR. Minimized Affinity: −9.08. Positions of the binding sites are outlined in dotted circles. **i** After treatment with ABP (10, 50, 100 μg/mL) for 24 h, cells were exposed to 50 ng/mL EGF for 10 min. Western blot analysis the effect of ABP on protein expressions of p-EGFR and EGFR. **j** Left panel: inhibition profiling was tested at the indicated concentrations of ABP with or without exposure to EGF in A549 cells. Right panel: The invasion ability of A549 and PC-9 cells in untreated, EGF (20 ng/mL) treated, and EGF (20 ng/mL) plus indicated concentrations of ABP-treated groups was assessed by transwell analyses. **k** Left panel: A, Photography of lungs of the mice inoculated with LLC cells via hypodermic injection; B, HE staining of lung metastatic nodules, amplification ×10. Right panel: the number of tumor metastatic nodules in the lungs of the mice. Data represented the mean ± SD, *n* = 8; ***P < 0.001 compared to the control group. **l** The possible mechanisms by which ABP inhibits cancer metastasis. ABP binding to extracellular domain of EGFR inhibits its activation and downstream signaling pathways, and blocks EMT process including inhibition of Snail expression, downregulation of N-cadherin and matrix metalloproteinase-2/9 (MMP-2/9), and upregulation of E-cadherin. EGF or ABP interacts with sites in domain I and domain III of EGFR. Domain II is dimerization arm, which extends the dimerization arm to hold the body of the other. In the left panel, the EGF chains in the EGF•EGFR dimer (pdb id 1mox) complex are red. Domains I, II, III, and IV in the receptor in the dimer are colored dark purple, green, blue, and gray, respectively. In the right panel, ABP in the ABP•EGFR complex is dark orange and domains I, II, III, and IV are colored dark purple, green, blue, and gray, respectively. The active kinases and downstream molecules are in red dotted square box; the inactive kinases in right panel are in gray dotted square box

To investigate whether ABP could suppress EMT-related molecules in NSCLC cells, we examined the expression of the epithelial makers E-cadherin and β-catenin, the mesenchymal markers N-cadherin and vimentin, and EMT-related transcription factor Snail by western blotting and quantitative reverse transcriptase PCR (qRT-PCR) assays. As shown in Fig. 1d and Supplementary Fig. S3a, b, the expressions of E-cadherin and β-catenin were dramatically increased, whereas the expressions of N-cadherin, vimentin, and Snail were significantly decreased by ABP treatment. Besides, the mRNA expression of E-cadherin was upregulated, whereas the mRNA expressions of N-cadherin and vimentin were downregulated (Supplementary Fig. S3c). Immunofluorescence analysis further verified that ABP reduced vimentin expression (Fig. 1e). We then assessed the effects of ABP on the protein and mRNA expressions of invasive-related molecules MMP-2 and MMP-9 by western blotting and qRT-PCR assays, respectively. ABP significantly inhibited the expressions of MMP-2 and MMP-9 (Supplementary Fig. S3d–f). In brief, ABP interfered with the EMT and the invasive cascade, thereby preventing the lung cancer metastasis.

During tumor progression and metastasis, EGFR kinase and its downstream signaling pathways have been implicated in the cellular survival in adhesive cancer cells and EMT process.^4^ Therefore, we further examined the effect of ABP on the EGFR activity and its downstream signaling pathways. Western blot analysis showed that ABP significantly inhibited the EGFR, p-EGFR, and its downstream kinase (phosphatidylinositol 3-kinase/protein kinase B/Akt) expressions (Fig. 1f and Supplementary Fig. S3g–i). Immunofluorescence (Fig. 1g) and qRT-PCR analyses (Supplementary Fig. S3j) further verified that ABP decreased the expression of EGFR. In addition, the expression of sarcoma gene (*Src*) was significantly reduced with ABP treatment (Fig. 1f and Supplementary Fig. S3k). However, the expressions of phosphatase and tensin homolog deleted on chromosome ten were increased with ABP treatment (Fig. 1f and Supplementary Fig. S3k). Interestingly, the computational modeling analysis of ABP (its structure was characterized by fourier transform-infrared spectrum, gel permeation chromatography, and high-performance liquid chromatography; Supplementary Table S1 and Supplementary Fig. S4) binding to EGFR using the known structure of EGFR (Protein Data Bank, 1IVO) indicated the strong binding of ABP to EGFR domain. The modeling further showed that this putative binding domain was similar to EGF (Fig. 1h). In addition, surface plasmon resonance analysis demonstrated the direct binding between ABP and EGFR with equilibrium dissociation constant (*K*_D_) value of 645 μM (Supplementary Fig. S5). These results suggest that ABP interacts with EGF binding site and blocks the EGF-induced EGFR activation. To further confirm ABP and EGF could competitively bind to EGFR, we treated A549 and PC-9 cells with EGF in the presence and absence of ABP, and then evaluated the activity of EGFR. Consistent with the molecular docking analysis, ABP significantly inhibited the total and phosphorylated EGFR even in the presence of EGF (Fig. 1i). In addition, low concentration of ABP showed a greater inhibition of p-EGFR in the presence of EGF compared with that with no EGF treatment (Fig. 1j, left panel). Importantly, when treated with EGF and ABP, the cell adhesion (Supplementary Fig. S6b) and invasion (Fig. 1j right panel and Supplementary Fig. S6a) induced by EGF were totally reversed even at a low ABP concentration (10 μg/mL). However, the ability of ABP to inhibit cell adhesion and invasion was not observed in another NSCLC cell line H1975 cells, which is EGFR tyrosine kinase inhibitor-insensitive cell line (Supplementary Fig. S7). These data strongly suggest that ABP binds to EGFR competitively with EGF and blocks EGFR activation and EMT process.

To examine the chemoprevention effect of ABP in vivo, we used the LLC murine artificial pulmonary metastatic model whose subcutaneous carcinoma LLC tumors were surgically removed and lung metastasis is caused by blood CTC.^5^ The number of tumor nodules on the lung surface in the ABP-treated group was significantly reduced compared to the control group (Fig. 1k). The ratio of lung weight to body weight was significantly decreased in ABP-treated group (250 mg/kg) compared to the control group (Supplementary Fig. S8b). In addition, the HE staining further revealed that ABP therapy reduced lung metastatic lesions in the disseminated lung cancer model (Fig. 1k (B)). Meanwhile, ABP did not cause significant body loss (Supplementary Fig. S8a) or cellular damages in the heart, liver, spleen, and kidney (Supplementary Fig. S8c), demonstrating that ABP effectively suppresses metastasis of LLC cells to the lungs without additional toxicity. To further examine the safety of ABP, we administrated normal mice with different doses of ABP (0, 250, 1000 mg/kg) by gavage for 5 days. As shown in Supplementary Table S2 and Supplementary Fig. S8d, there were no obvious abnormalities in blood biochemistry and histological examination. Furthermore, the EGFR (Supplementary Fig. S8e, f) and vimentin (Supplementary Fig. S8e, g) expressions were significantly downregulated in the ABP-treated group.

Accumulating evidence reveals that the embryo implantation into the endometrium and CTCs adhesion–invasion to vascular endothelium are similar. Thus, those traditional contraceptives with safe anti-implant ability could be used as cancer metastasis chemoprevention agents. The roots of *A. bidentata* have been used for abortion in China for a long history. In the present study, we tried to find the safe and effective pre-metastatic chemoprevention from these TCM. We here demonstrate that ABP, which was isolated from *A. bidentata*, is a natural EGFR inhibitor that can safely and effectively prevent cancer metastasis via blocking cell adhesion to vascular endothelium and EMT process. Importantly, ABP acts as a competitive inhibitor and accommodates between domains I and III of EGFR (Fig. 1h), thereby inhibiting EGF-induced dimerization of EGFR and activation of downstream signaling pathways by directly blocking ligand binding (Fig. 1l). These findings suggest the clinical implication of ABP for EGFR-related NSCLC cancer as a metastasis chemoprevention agent. This study sets up a model to screen and repurpose many safe and effective TCM for cancer metastasis chemoprevention.

## Supplementary information

Supplementary files
