# Author Correction: *Achyranthes bidentata* polysaccharide can safely prevent NSCLC metastasis via targeting EGFR and EMT

**DOI:** 10.1038/s41392-020-00322-4

**Published:** 2020-10-13

**Authors:** Chunlian Zhong, Jingyi Yang, Yusheng Lu, Huanzhang Xie, Shengyi Zai, Chen Zhang, Zhiying Luo, Xuanchen Chen, Xuanmo Fang, Lee Jia

**Affiliations:** 1 Institute of Oceanography, Minjiang University, Fuzhou, Fujian 350108 China; 2 Cancer Metastasis Alert and Prevention Center, College of Chemistry, Fujian Provincial Key Laboratory of Cancer Metastasis Chemoprevention and Chemotherapy, Fuzhou University, Fuzhou, Fujian 350116 China; 3 Fujian Provincial People’s Hospital Affiliated to Fujian University of Traditional Chinese Medicine, Fuzhou, 350004 China

**Keywords:** Metastasis, Lung cancer

Correction to: *Signal Transduction and Targeted Therapy* 10.1038/s41392-020-00289-2, published online 31 August 2020

In the process of collating the raw data, the authors noticed one inadvertent mistake in Fig. 1 that need to be corrected.^1^ The correct data are provided as follows. The key findings of the article are not affected by these corrections.

**Fig. 1j:**
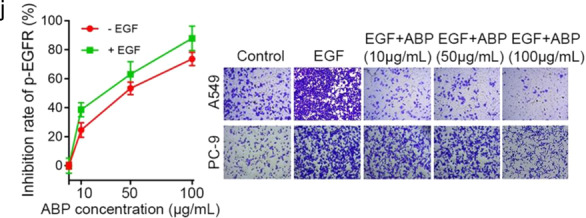
Left Panel: inhibition profiling was tested at the indicated concentrations of ABP with or without exposure to EGF in A549 cells. Right panel: The invasion ability of A549 and PC-9 cells in untreated, EGF (20 ng/mL) treated, and EGF (20 ng/mL) plus indicated concentrations of ABP-treated groups was assessed by transwell analyses

In the right panel of Fig. 1j, the represented image showing the invasion ability of A549 cells in EGF groups was made a mistake when pasted the picture, which is an inadvertent mistake. After checked the original data, the corrected version of the figure is shown below.
