# Supplementary material for: Achyranthes bidentata polysaccharide can safely prevent NSCLC metastasis via targeting EGFR and EMT
Source: Signal Transduct Target Ther. 2020 Aug 31;5:178. doi: 10.1038/s41392-020-00289-2 (PMC7459280; doi:10.1038/s41392-020-00289-2)
Supplement: Supplementary file 1 — Supplementary files [file 41392_2020_289_MOESM1_ESM.docx]

**Supplemental Materials**

***Achyranthes bidentata* polysaccharide can safely prevent NSCLC metastasis via targeting EGFR and EMT**

Chunlian Zhong^1#^, Jingyi Yang^2#^, Yusheng Lu^1#^, Huanzhang Xie^1#^, Shengyi Zai^2^, Chen Zhang^1^, Zhiying Luo^1^, Xuanchen Chen^3^, Xuanmo Fang^3^, Lee Jia^1,2^*

^1^Institute of Oceanography, Minjiang University, Fuzhou, Fujian 350108, China; ^2^Cancer Metastasis Alert and Prevention Center, College of Chemistry; Fujian Provincial Key Laboratory of Cancer Metastasis Chemoprevention and Chemotherapy, Fuzhou University, Fuzhou, Fujian 350116, China;

^3^Fujian Provincial People’s Hospital Affiliated to Fujian University of Traditional Chinese Medicine, Fuzhou, 350004, China

^#^These authors contributed equally to this work.

*Corresponding Author: Lee Jia ([pharmlink@gmail.com](mailto:pharmlink@gmail.com) or [cmapcjia1234@163.com](mailto:cmapcjia1234@163.com))

**Supplementary information include:**

Materials and Methods

Tables S1 to S2

Figures. S1 to S8

**Materials and Methods**

***Cell lines and cell culture***

Human non-small cell lung cancer cell lines A549 cells, PC-9 cells, H1975 cells and Lewis lung carcinoma (LLC) cells were obtained from the Chinese Academy of Sciences Cell Bank of Type Culture Collection (Shanghai, China). Human pulmonary microvascular endothelial cells (HPMECs) were obtained from Shanghai Zhong Qiao Xin Zhou Biotechnology Co., Ltd. The A549 cells, PC-9 cells and LLC cells were cultured in F-12k medium (obtained from HyClone)，RPMI medium (obtained from HyClone) and DMEM medium (obtained from HyClone) supplemented with 10% fetal bovine serum (FBS, obtained from Gibco), 100 units/mL penicillin, and 100 μg/mL streptomycin, respectively. H1975 cells were cultured in RPMI medium (obtained from HyClone) supplemented with 10% fetal bovine serum (FBS, obtained from Gibco), 0.2% Glucose (obtained from Sinopharm), 1% Sodium Pyruvate (obtained from Leagene), 100units/mL penicillin, and 100 units/mL streptomycin. The HPMEC cells were maintained in 1% gelatin-coated tissue culture flasks containing M199 medium (obtained from Gibco) supplemented with 20% FBS, 8 units/mL heparin, 100μg/mL ECGS, 100units/mL penicillin and 100units/mL streptomycin, and discarded after 6 passages. The cells were maintained in a humidified incubator with 5% CO_2_ at 37 °C***.*** The A549 cells, PC-9 cells, H1975 cells and LLC cells used in our experiments are less than fifty generation.

***Plant material and isolation of ABP***

A. *bidentata* roots were purchased from FuJian China, the place of origin was HeNan and the production lot number was 20150813. The roots of A. *bidentata* were pulverized and pretreated twice with 75% ethanol to remove some impurities. The pretreated dry powder was extracted three times (3h each time) with ultra-pure water. The extract solution was centrifuged (12000rpm for 30min), then the supernatant was separated from insoluble residue with gauze. The extract was degreased using the Sevag method and was dialyzed in cellulose membrane. Subsequently, the extract solution was precipitated by adding twice the volume of ethanol. The precipitate was collected by centrifugation (12000rpm for 30min), then dissolved in ultra-pure water (resistivity ＞ 18m Ω·cm), and lyophilized. To obtain the purified ABP，the sample was further dissolved in ultra-pure water and fractionated on a DEAE-cellulose 52 column (Ø 2.6 × 40cm).

***Phytochemical analysis***

According to the previously recorded methods[^1^](#_ENREF_1) [^2^](#_ENREF_2), the existence of various active constituents in the extract were tested by color reaction. Molish test and Fehling test were performed for detecting the presence of carbohydrates and glycosides. Libermann-Burchard test was performed for detecting the presence of phytosterols and saponins. Salkowski reaction was performed for detecting the presence of indoles. Hager test and Dragendorff test were performed for detecting the presence of alkaloids.

Fourier transform-infrared spectrum (FT-IR) analysis was acquired by using an intelligent AVATAR-360 FTIR (Thermo Nicolet Corporation, USA). The dry sample and potassium bromide were fully grinded and mixed, and the mixture was put into the mold for scanning. The scanning range was 4000-400 cm^-1^.

The molecular weight of sample was determined by gel permeation chromatography using Viscotek TDA305 GPC system (Malvern, UK) equipped with a Guard +A6000M × 1 column, a refractive index detector (RID), a right-angle light scattering detector (RAILS) and a viscosity detector (DP). The sample was dissolved in ddH_2_O of 0.1 M NaNO_3_ (3 mg/mL). The mobile phase was water containing 0.02% NaN_3_; the flow rate was 0.7 mL/min; the injection volume was 100μL; the column temperature was set at 35℃.

The monosaccharide composition of the sample was determined by high performance liquid chromatography (Waters 2695). The sample and monosaccharide standards were hydrolyzed with trifluoroacetic acid (TFA). Hydrolyzed sample was dissolved in 1-phenyl-3-methyl-5-pyrazolone (PMP) solution and NaOH solution for derivatization, and then used a Thermo ODS-2 C18 column (4.6 × 250mm, 5μm) maintained at 25℃ with isocratic elution of 0.1 M pH 6.8 PBS-acetonitrile (82:18, v:v) to analysis. The flow rate was 1.0mL/min; the injection volume was 10μL; the detection wavelength was set at 245nm.

***Reagents and antibodies***

Human interleukin-1 beta (IL-1β) (#8900) was purchased from Cell Signaling Technology, Inc. E-cadherin (1:1000, 3195), N-cadherin (1:1000, 13116), β-catenin (1:1000, 8480), vimentin (1:1000, 5741S), snail (1:1000, 3879), MMP2 (1:1000, 40994), MMP9 (1:1000, 13667), p-Akt (1:2000, 4060) and p-PI3K (1:1000, 17366) were obtained from Cell Signaling Technology. PI3K (1:100, ab70912), Akt (1:500, ab18785), Src (1:1000, ab47405), PTEN (1:10000, ab32199), and β-actin (1:5000, ab8227) were obtained from Abcam. EGFR (1:1000, A11575), p-EGFR (1:1000, AP0301) were obtained from ABclonal. Goat anti-rabbit IgG (H+L) horseradish peroxidase secondary antibody (111-035-144) was obtained from Jackson. 549-conjugated goat anti-rabbit IgG (H＆L) secondary antibody dylight (611-142-002) was obtained from Rockland. Matrix was obtained from BD Biocoat ^TM^.

***Animals and ethics statement***

Female C57BL/6 mice (20-22g), aged 4‐6 weeks, were obtained from Shanghai SLAC Laboratory Animal Co., Ltd. These mice were fed pellet food and water in a constant comfortable environment at room temperature. All mice were anesthetized by i.p. injection of sodium pentobarbital (0.7mg/20g mouse). All studies involving animals were conducted in accordance with National Institutes of Health guide for the care and use of Laboratory animal regulations.

***Cytotoxicity assay in vitro***

The cytotoxicity of ABP was investigated by the 3-(4, 5-dimethylthiazol-2-yl)-2, 5-diphenyltetrazolium bromide (MTT) assay. When incubated with live cells, MTT is reduced by the mitochondria to purple formazan crystals, which can be quantified at 570nm using an infinite M200 Pro microplate reader. Briefly, A549 cells, PC-9 cells, HPMECs cells, H1975 cells and LLC cells (1×10^4^ per well/100μL) were seeded into 96-well plates containing F-12k, RPIM, M199 and DMEM medium, respectively. After overnight incubation, the medium was replaced with various concentrations (0-100μg/mL) of ABP for 24 h. Finally, MTT solution (5 mg/mL) was added, and the cells were incubated in the medium without phenol red and serum for another 4h. The MTT formazan product was dissolved in 100μL DMSO and shaken for 10min. The optical density (OD) at 570nm was recorded by using an infinite M200 Pro microplate reader (Tecan, Switzerland). Each concentration was repeated at least three times. The relative viability rate (%) = (A-A_0_)/(A_1_-A_0_) × 100%.

A: OD value of the experimental group; A_0_: OD value of the parallel solvent control group; A_1_: OD value of the blank control group.

***Cells apoptosis assay***

Cells apoptosis was detected by using an Annexin V-FITC/PI Staining Kit (Signalway Antibody, CA001-2). Cells (4×10^5^ per well) were seeded into 6-well plates and incubated with various concentrations (0, 10, 50, 100μg/mL) of ABP for 24 h. Then, cells were washed, trypsinized, harvested, suspended in binding buffer and stained with Annexin V-FITC and PI following the manufacturer’s instructions. Suspended cells were incubated with Annexin-V and PI for 15 min at 25°C in the dark. After washed by PBS, the samples were immediately analyzed by flow cytometry (BD Biosciences).

***Adhesion assay***

The adhesion of NSCLC cells to the HPMECs was carried out by the fluorescence microscope photographing method. HPMEC cells (1×10^5^ per well) were seeded into 24-well plates containing M199 medium (10% FBS), and cultured to form endothelial monolayer, followed by stimulation with 500μL of 1ng/mL IL-1β for 4h. Simultaneously, A549 cells were stained with rhodamine-123 for 15min. Then, the cells (5×10^4^ per well) were re-suspended in serum-free, phenol red-free RPMI 1640 media with different concentrations of ABP (0, 10, 50, 100μg/mL) and seeded to the wells covered with HPMECs. After co-incubation for 1h, cells were gently washed three times with phosphate buffered saline (PBS) to remove the non-adherent cells. Each well randomly selected 10 visual fields and taken pictures using an inverted fluorescence microscope. Adherent cells were counted by using Image J software. Mean inhibition of adhesion for 10 visual fields was calculated by the following equation: Relative adhesion rate (%) = (Number of adherent cells in experimental group/number of adherent cells in control group) × 100%.

The adhesion of NSCLC cells to the gelatin was assessed according to our previous described method[^3^](#_ENREF_3). Each well of a 96-well plate was covered with 2μg of gelatin and dried overnight in a biological safety cabinet at room temperature. Then the excess gelatin was washed with PBS and blocked with 100μL of 1% bovine serum albumin (BSA, Sigma) solution at 37°C, 5% CO_2_ for 1h. Cells (1×10^4^ per well/100μL) were suspended in medium containing the indicated concentrations of ABP (0, 10, 50, 100μg/mL) and added to each well for 1h. Finally, non-adherent cells were washed three times with PBS and removed. And then the MTT solution was added and the absorbance values were measured as described above. The adhesion rate was calculated by the following equation: Relative adhesion rate (%) = (OD value of treatment group- OD value of solvent blank group)/(OD value of control group - OD value of solvent blank group) × 100%.

***Wound healing migration assay***

A wound-healing migration assay was performed to analyze cell migration *in vitro*. NSCLC cells were seeded into a 24-well plate at a density of 1×10^5^ cells per well. After 24h, the cell monolayers were scratched gently by using a sterile pipette tip of 10μL, which were washed three times with PBS and cultivated in serum-free medium containing different concentrations (0, 10, 50, 100μg/mL) of ABP. The same wounded areas were imaged at 0 and 24 h and quantified using Image J software. Comparison of the initial distance and the final distance of the marked areas was used to evaluate the cell migration ability.

***Invasion assay***

The inhibitory effect of ABP on the invasive ability of NSCLC cells was analyzed by the transwell invasion assay. Brieﬂy, the transwell culture chambers (24-well, 8μm pore size, Costar, Corning Incorporated, USA) were placed in a 24-well plate and the upper chambers were coated with matrix and air-dried. After 24h, 5×10^4^ cells were suspended in 500μL of serum-free medium containing determined concentrations of ABP and placed into the upper chamber of the wells. The lower chambers were added with 500μL of medium containing 20% FBS. After incubation for 48h, the cells in the upper chambers were carefully wiped off with a cotton swab. Cells invaded through the matrix were fixed with 4% (w/v) paraformaldehyde and stained with crystal violet. The invading cells were counted and photographed (five random fields) under an optical microscope (Zeiss, Germany).

***Western blotting analysis***

NSCLC cells were seeded into a 6-well plate (3×10^5^ per well) and incubated in medium for 24h, then treated with various concentrations of ABP (0, 10, 50, 100μg/mL) for 24h. The cells were washed three times with pre-cooled PBS and lysed with RIPA buffer (Roche) on ice. The equal amounts of denatured protein samples were separated on 6% to 10% (w/v) sodium dodecyl sulfate (SDS)-polyacrylamide gel, and transferred to polyvinylidene ﬂuoride (PVDF) membranes (Bio-Rad). The membranes were blocked in 5% BSA at room temperature for 1h and incubated with the primary antibodies E-cadherin, N-cadherin, β-catenin, vimentin, snail, MMP2, MMP9, EGFR, p-EGFR, PI3K, p-PI3K, Akt, p-Akt, Src, PTEN or β-actin overnight at 4°C. And then, the membranes were incubated with horseradish peroxidase-linked secondary antibody at room temperature for 1h. After washed with TBST, the membranes were exposed to the ChemiDoc XRS System (Bio-Rad) to detect the expressions of the target proteins, which were enhanced by using the ECL Kit (Wanlei Biotechnology, China) and quantified with Image Lab software (Bio-Rad), with normalization to β-actin levels.

***Immunoﬂuorescence microscopy***

Cells (2×10^4^ per well) were seeded on 12-well coverslips overnight and then cultured with or without 100μg/mL ABP for 24h. The cells were washed three times with PBS and fixed with 4.0% (w/v) paraformaldehyde for 10min, permeabilized with 0.1% (v/v) Triton X-100 in PBS for 5min, blocked with fresh 10% goat serum for 30min to reduce non-specific background. The cells were stained with anti-EGFR antibody overnight at 4°C or incubated with a specific antibody against vimentin overnight at 4°C. The cells were then rewashed, and incubated with 549-conjugated goat anti-rabbit IgG for 30min at 37℃. Finally, the cells were washed and counterstained with DAPI for 10min and then analyzed by the Leica SP8 confocal laser scanning microscope (Leica) at 630× magnification under oil immersion.

***Immunohistochemistry***

The paraffin-embedded tissues were cut into 6μm sections. After deparaffinization and rehydration, endogenous peroxidase was blocked by incubation in 3% H_2_O_2_ for 10minutes. Followed by antigen retrieval and blocked by 10% bovine serum albumin, the lungs sections were incubated with anti‐EGFR antibody (1:100, ABclonal, A11575), anti‐vimentin antibody (1:200, Cell Signaling, 5741S) overnight at 4°C. Afterwards, the sections were washed 3 times in PBS, followed by incubation with horseradish peroxidase‐conjugated secondary antibodies (Jackson, 1: 500) for 1h at room temperature. The immune reaction was performed using a DAB Kit (MXB, China) under microscope. The sections were counterstained with hematoxylin, gradient alcohol dehydration and mounted. Stained tissues were visualized under a light microscope at 40× magnification.

***Total RNA isolation and*** ***quantitative real-time PCR***

The total RNA of A549 cells was extracted with Trizol reagent (Invitrogen). And then the purified RNA was used to synthesize cDNA with the PrimeScript^®^ RT Reagent Kit (Takara, Japan) according to the manufacturer’s instructions. The PCR amplification was carried out with cDNA (＜100ng) in 25μL reaction volume using SYBR^®^ Premix Ex Taq™ PCR Kit (Takara, Japan) on the CFX96^TM^ Real-Time PCR Detection Systems (Bio-Rad). The following gene-specific primer sequences were used for real-time PCR analysis:

EGFR-forward (5’-CTTGCAGCGATACAGCTCAG-3’), EGFR-reverse

(5’-AGGAGGTTGAGGAGCAGGAC-3’);

E-cadherin-forward (5’-AACTCCAGGCTAGAGGGTCA-3’), E-cadherin-reverse (5’-TCACAGGTGCTTTGCAGTTC -3’);

N-cadherin-forward (5’-ATGAAGAAGGTGGAGGAGA-3’), N-cadherin-reverse (5’- AGATCGGACCGGATACT-3’);

vimentin-forward (5’-AGGACTCGGTGGACTTCTCG-3’), vimentin-reverse (5’-AGCGCACCTTGTCGATGTAG-3’);

MMP-9-forward (5’- TTGACAGCGACAAGAAGTGG-3’), MMP-9-reverse (5’-GCCATTCACGTCGTCCTTAT-3’);

MMP-2-forward (5’-TCTCCTGACATTGACCTTGGC-3’), MMP-2-reverse (5’- CAAGGTGCTGGCTGAGTAGATC -3’) and β-actin-forward (5’- GTTGCTATCCAGGCTGTG -3’), β-actin-reverse (5’-TGATCTTGATCTTCATTGTG-3’). Target mRNA expression levels were normalized with β-actin mRNA and the relative gene expression was calculated using the 2^-△△Ct^ method. Results were expressed as the mean ± standard deviation of three independent experiments.

***Modeling of interactions between ABP and EGFR***

The crystal structure for EGFR was obtained from Protein Data Bank (PDB ID: 1IVO), and the 3D coordinates for ABP were generated by Open Babel. Docking poses of ABP and EGFR were refined further by using SMINA program[^4^](#_ENREF_4).

***Binding affinity between ABP and EGFR***

The binding affinity of ABP to recombinant EGFR was determined by surface plasmon resonance (SPR) analysis performed using MP-SPR Navi™200 OTSO (BioNavis). Recombinant EGFR purchased from novoprotein (C154) in sodium acetate solution (10mM, pH 3.8) was immobilized on biosensor chips with a standard amine coupling kit (GE healthcare) as activator. Different concentrations of ABP (0, 5, 10, 20, 50, 100, 250, 500, 1000μg/mL) were flowed over the EGFR surface successively in PBS at 25℃. 10 mM glycine-HCl (pH 3.0) was used to regenerate the sensor chip after each injection cycle. The association and dissociation phases of binding curves were analyzed by using BIA evaluation kinetics software, and the affinity and dissociation constants were calculated.

***Development of lung metastasis induced by LLC cells***

The right legs hair of female C57BL/6 immunocompetent mice were shaved with a razor, and then each mouse was given a subcutaneous injection of 1×10^6^ LLC cells in 0.1 ML PBS. About 20 days after the LLC inoculation, the tumor size reached 1- 1.5cm^3^ and the tumors were surgically removed [^5^](#_ENREF_5). The mice were randomly divided into three groups (n*=*8 in each group), each was given the oral ABP suspended in PBS at 0, 25 and 250mg/kg for 30 days. Body weight was measured and recorded every two days. After the treatment, the mice were sacrificed. The lungs, heart, livers, spleen and kidneys were dissected, washed with PBS and fixed in 4% (v/v) paraformaldehyde. The number of surface pulmonary metastasis nodules in each group was evaluated. All tissues were paraffin embedded followed by H&E staining.

***Statistical analysis***

Data represented mean ± standard deviation (SD). The experimental data were analyzed statistically by one-way analysis of variance (ANOVA) by using Prism 7.0 (Graphpad Software, Inc) statistical software. *P*< 0.05 was considered statistically significance and *P*< 0.01 was considered extremely statistically significance. **P< 0.05; **P< 0.01; ***P< 0.001.*

**Data availability**

The datasets generated and/or analyzed during the current study are available from the corresponding author upon reasonable request.

**Author contributions**
C.Z., Y.L., and L.J. conceived and designed the experiments; H.X., J.Y., S.Z. and Z.L. performed the experiments; C.Z., X.C., J.Y. and X.F. performed the data analyses; C.Z. and L.J. drafted and revised the manuscript; L.J. finalized the manuscript.

**References**

1 Venkateswarlu, V., Kokate, C. K., Peddanna, G., Veeresham, C. & Rambhau, D. Pharmaceutical investigations on salacia macrosperma-1. *Ancient science of life* **9**, 215-219 (1990).

2 Krishnaveni, A. & Thaakur, S. R. Pharmacognostical and preliminary phytochemical studies of achyranthes aspera linn. *Ancient science of life* **26**, 1-5 (2006).

3 Zheng, G. *et al.* Corrigendum to 'Metapristone suppresses non-small cell lung cancer proliferation and metastasis via modulating RAS/RAF/MEK/MAPK signaling pathway' [Biomed. Pharmacother. 90 (2017) 437-445]. *Biomedicine & pharmacotherapy = Biomedecine & pharmacotherapie* **98**, 925-926, doi:10.1016/j.biopha.2018.01.091 (2018).

4 Koes, D. R., Baumgartner, M. P. & Camacho, C. J. Lessons learned in empirical scoring with smina from the CSAR 2011 benchmarking exercise. *Journal of chemical information and modeling* **53**, 1893-1904, doi:10.1021/ci300604z (2013).

5 Su, X. *et al.* A simple and effective method for cancer immunotherapy by inactivated allogeneic leukocytes infusion. *International journal of cancer* **124**, 1142-1151, doi:10.1002/ijc.24045 (2009).

**Table. S1**

**Supplementary Table. S1.** The results of chemical identification.

| **Fast assay** | **Resulted color** | **Test color** |
| --- | --- | --- |
| Molish | Purple ring | Carbohydrates |
| Fehling | Red precipitation | and glycosides |
| Libermann-Burchard | No change | No sterols and saponins |
| Salkowski reaction | No change | No Indoles |
| Hager | No change | No alkaloids |
| Dragendorff | No change |  |

**Table. S2**

| **Supplementary Table. S2.** Blood chemistry of mice administered with oral ABP for 5 days. | | | | |
| --- | --- | --- | --- | --- |
|  |  | **ABP (mg/kg/day)** | |  |
| **Item** | **Control (n=20)** | **250**  **(n=10)** | **1000**  **(n=10)** | **Reference** |
| AST (U/L) | 167.8±36.88 | 177.2±40.82 | 230.65±25.18 | 55-352 |
| ALT (U/L) | 44.3±34.309 | 65±15.302 | 74.45±29.421 | 40-131 |
| BUN(mg/L) | 245.4±26.3 | 240.1±25.19 | 255.6±38.9 | 70-310 |
| RBC(M/µL) | 10.533±0.3478 | 10.79±0.3155 | 10.44±0.9304 | 8.16-11.69 |
| WBC(K/µL) | 7.133±1.579 | 6.942±1.52 | 6.829±1.559 | 5.69-14.84 |
| HGB (g/L) | 150.1±0.4767 | 152.9±0.4677 | 150.1±1.21 | 124-189 |
| HCT (%) | 50.16±1.532 | 51.25±1.617 | 49.4±4.402 | 43-67 |
| PLT (K/µL) | 938.1±200.5 | 1075±178 | 1102±222.1 | 476-1611 |
| MCV (fL) | 57.66±0.5679 | 57.48±1.319 | 57.3±0.7572 | 50.8-64.1 |
| MPV (fL) | 5.795±0.4249 | 5.6±0.1886 | 5.73±0.1494 | 4.6-5.8 |
| MCH (pg) | 14.27±0.2003 | 14.17±0.3683 | 14.36±0.2875 | 13-17.6 |
| MCHC(g/L) | 299.3±0.3614 | 298.4±0.1647 | 304.1±0.4807 | 239-331 |
| Aspartate aminotransferase (AST), alanine transaminase (ALT), and blood urea nitrogen (BUN). Red blood cell (RBC), white blood cell (WBC), hematoglobin (HGB), hematocrit (HCT), blood platelet (PLT),erythrocyte mean corpuscular volume (MCV), mean platelet volume (MPV), mean hemoglobin concentration (MCH), mean corpsular hemoglobin (MCHC). Reference ranges of hematology data for healthy female mice were obtained from Charles River Laboratories. | | | | |

**Fig. S1**

**
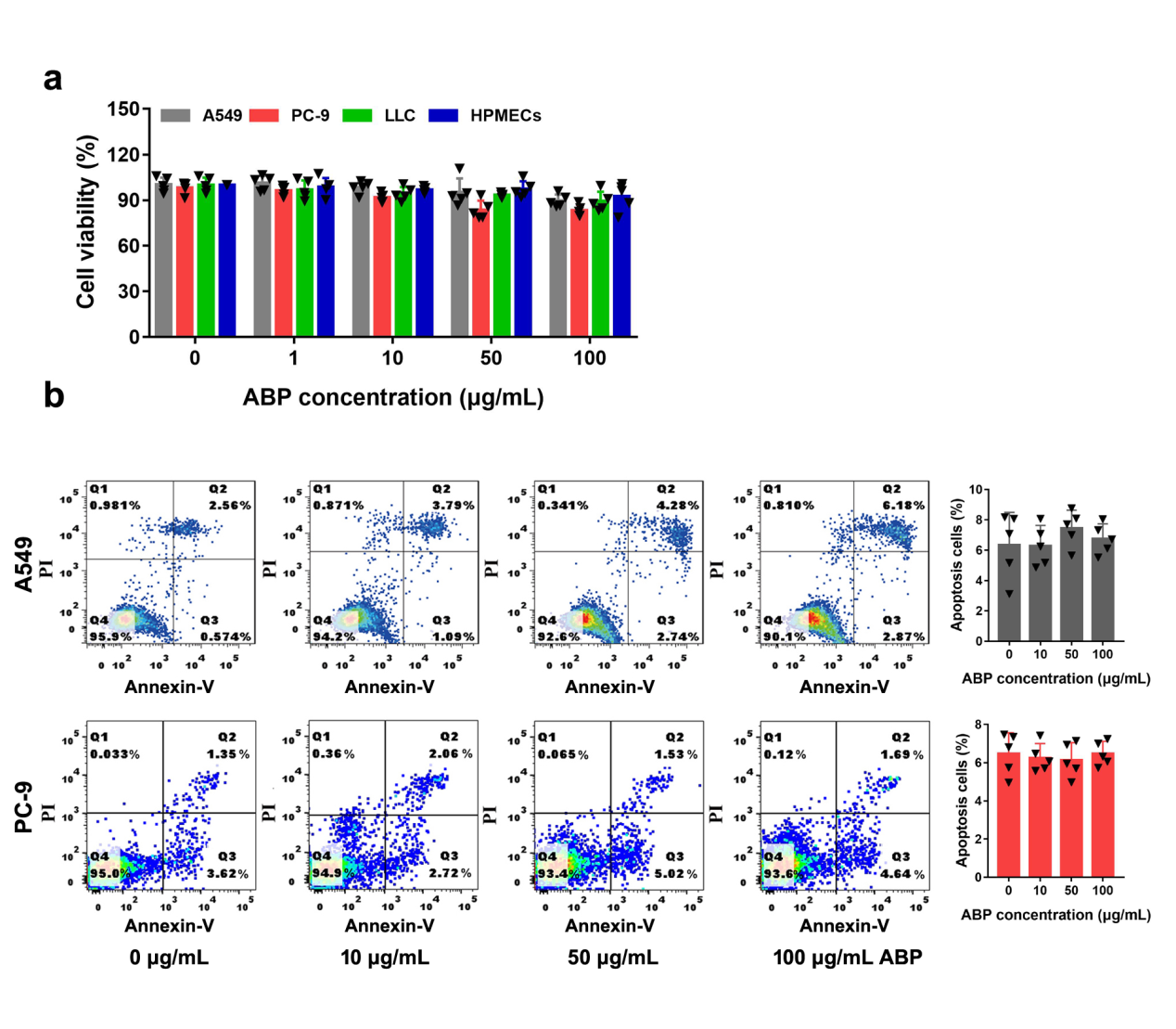
**

**Supplementary Fig. S1. Cytotoxicity of ABP *in vitro*.** (a) Cells (1×10^4^ per well) were seeded into 96-well plates. After incubation with ABP for 24h, cell viability was measured by MTT assays. (b) Cells (4×10^5^ per well) were seeded into 6-well plates and incubated with different concentrations of ABP for 24h. The apoptosis of A549 and PC-9 cells was analyzed by flow cytometry, and the quantitative analysis is shown in the right panel. Q2 quadrant represents the late stage apoptotic cells and Q3 quadrant represents the early stage apoptotic cells. Data represented the mean ± SD, n = 5.

**Fig. S2**


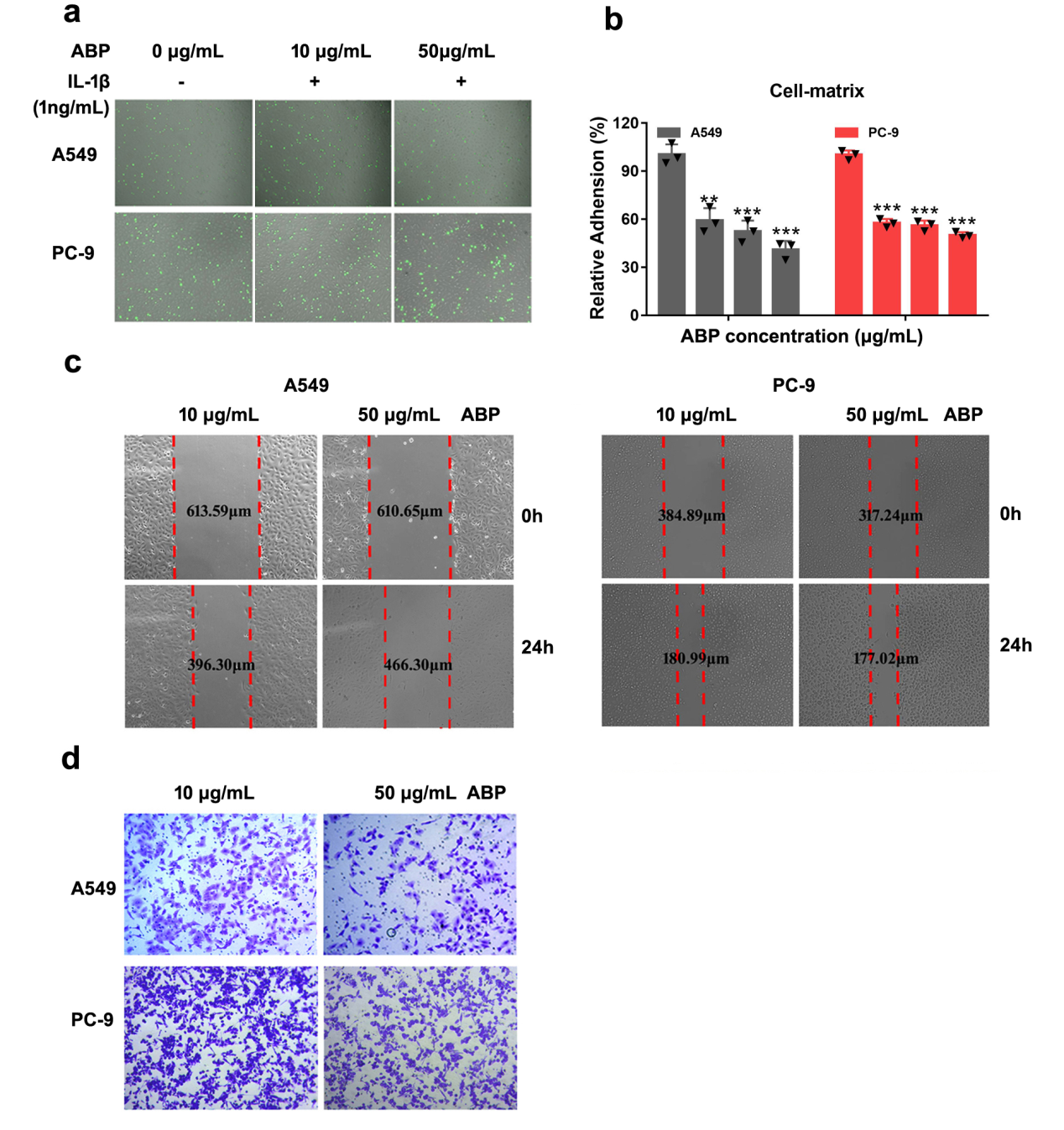


**Supplementary Fig. S2. Effects of ABP on adhesion and metastasis of A549 and PC-9 cells.** (a) Fluorescence microscopic images show that ABP at different concentrations inhibited adhesion of rhodamine-123 labeled A549 and PC-9 cells to the HPMECs monolayers stimulated by IL-1β (1ng/mL). The green fluorescing dots represent adhesion of A549 or PC-9 cells to HPMECs. (b) Quantitative analysis of the inhibition of ABP on adhesion of A549 and PC-9 cells to cell-matrix. (c) Micrographs of A549 and PC-9 cells treated with ABP at 0 and 24h after monolayer wounding. (d) Representative images of the transwell invasion of A549 and PC-9 cells treated with ABP at different concentration for 48 h. Data represented the mean ± SD, n = 3; **P < 0.05, **P < 0.01, ***P < 0.001* compared to the control group.

**Fig. S3**

**
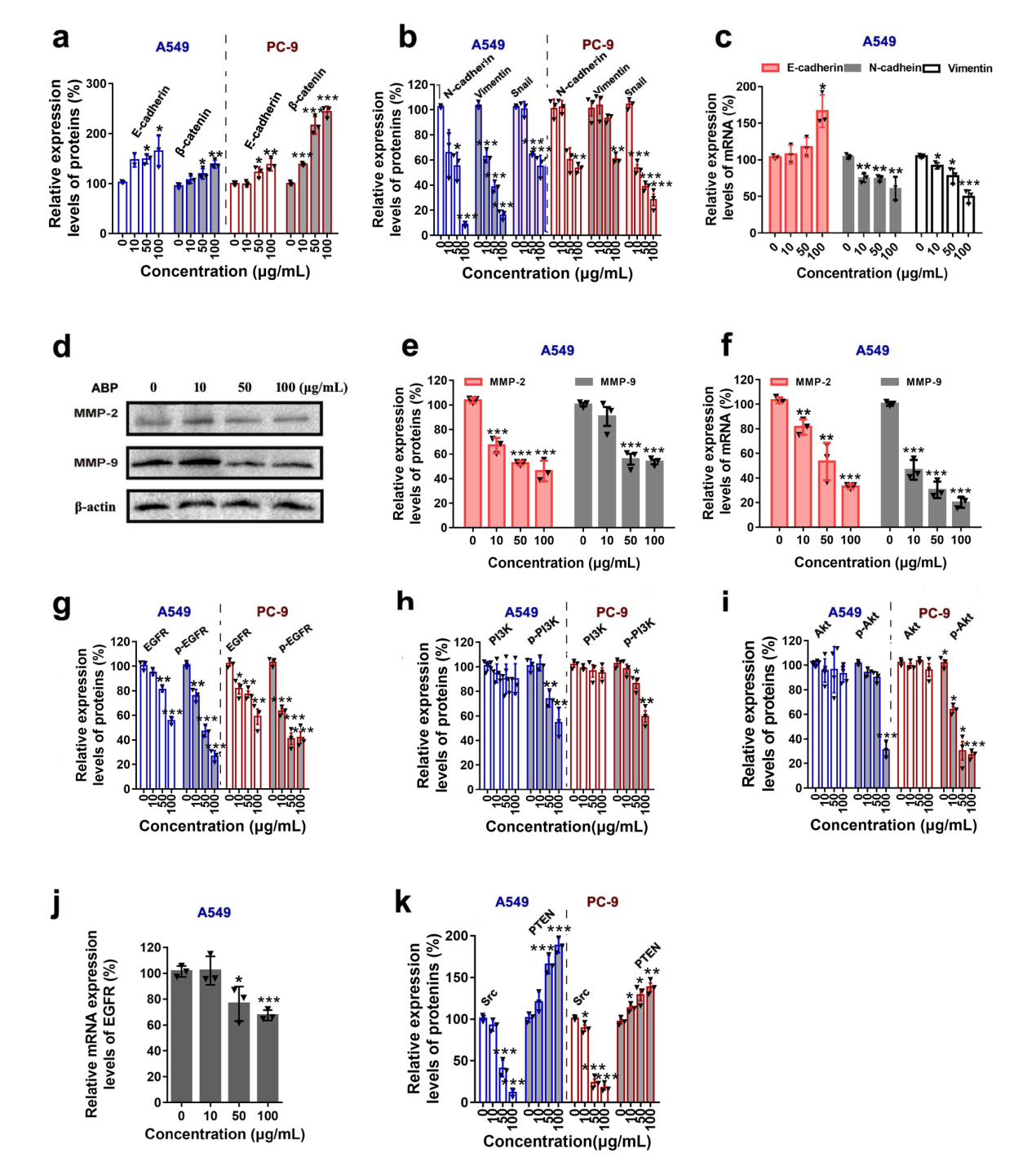
**

**Supplementary Fig. S3. Changes in EMT-related molecules, invasion molecules, EGFR and its downstream signaling pathways in A549 and PC-9 cells after 24-h ABP treatment.** (a-b) Quantitative analyses protein expressions of E-cadherin, β-catenin, N-cadherin, vimentin and Snail in A549 and PC-9 cells. (c) Real-time PCR showing that ABP significantly decreased the mRNA expressions of N-cadherin and vimentin, and increased the mRNA expression of E-cadherin in A549 cells. (d-e) Western blot analysis of MMP-2 and MMP-9 protein expressions in A549 cells treated with ABP for 24h. (f) Real-time PCR analysis of MMP-2 and MMP-9 in A549 cells. (g-i) Quantitative analysis of protein expressions of EGFR, p-EGFR, PI3K, p-PI3K, Akt and p-Akt in A549 and PC-9 cells treated with ABP for 24h. (j) Real-time PCR results indicated that ABP significantly decreased the mRNA expressions of EGFR. (k) Quantitative analyses of protein expressions of Src and PTEN in A549 and PC-9 cells treated with ABP for 24 h. Data represented the mean ± SD, n=3; **P < 0.05, **P < 0.01, ***P < 0.001* compared to the control group.

**Fig. S4**

**
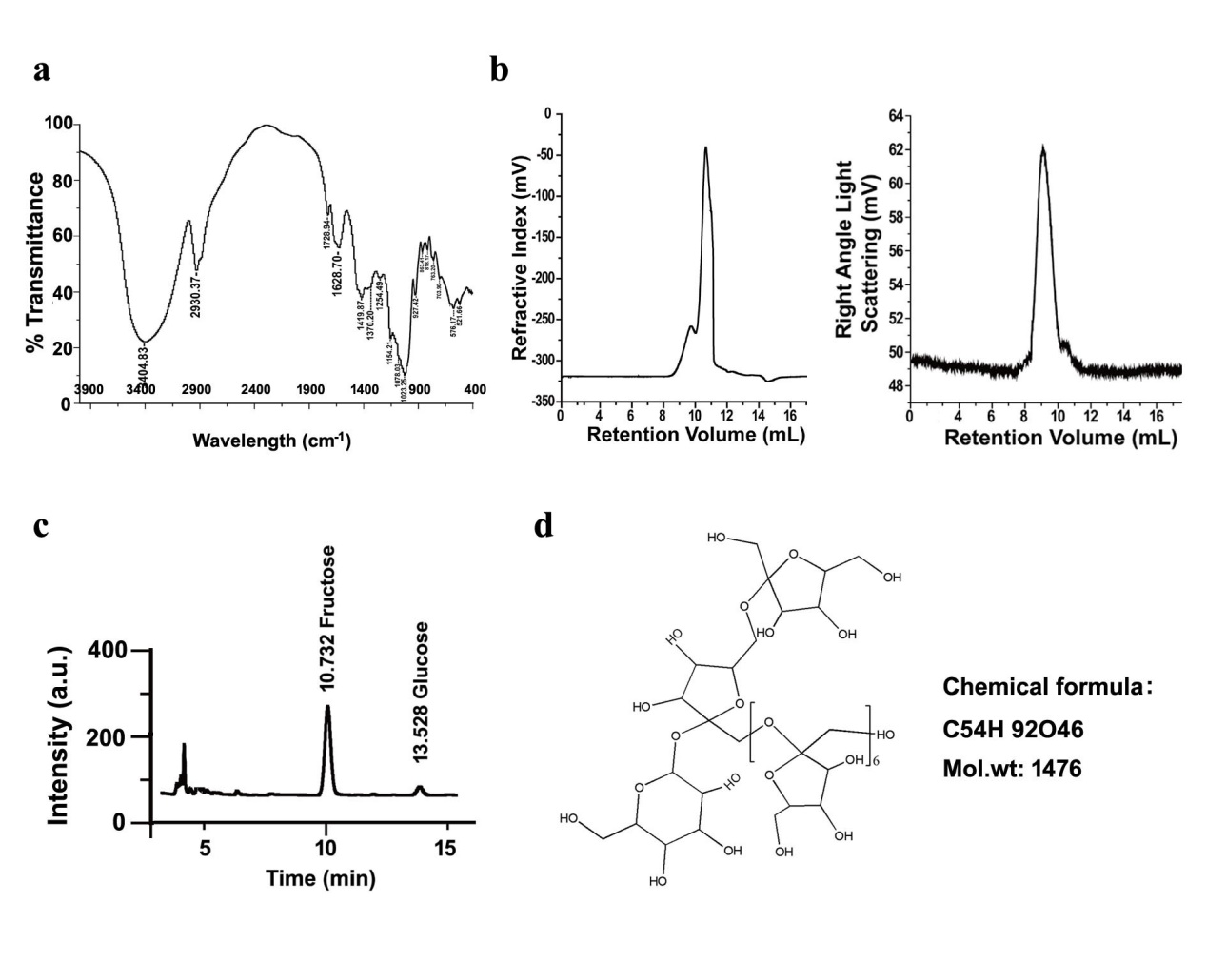
Supplementary Fig. S4.** **Phytochemical and composition analyses of ABP.** (a) FT-IR spectral analysis of ABP showing its characteristic absorption peaks. A strong and wide absorption peak at 3404.83 cm^-1^ represents the stretching vibration of O-H. The small absorption peak at 2930.37 cm^-1^ represents the stretching vibration of C-H in the methyl (-CH3) and methylene (-CH2) groups. The absorption peaks of 1728.94 cm^-1^ and 1628.70 cm^-1^ are the stretching vibration of C=O, which are the characteristic absorption peaks of glucuronic acid. The absorption peaks of 1419.87 cm^-1^, 1370.20 cm^-1^ and 1254 cm^-1^ are caused by the variable angle vibration of C-H, which combine with the peak at 2930.37 cm^-1^, illustrate the presence of sugar ring. The absorption peaks of 1154.21 cm^-1^, 1078.01 cm^-1^ and 1023.25 cm^-1^ between 1200-1000 cm^-1^ are the stretching vibration of C-O-C and C-O-H, indicate existence of monosaccharides as the form of pyranoside; the absorption peak at 927.42 cm^-1^ represents the stretching vibration of the asymmetric ring in the pyranoside. (b) GCP analysis shows that the elution mainly contains a molecule of 1476 molecule weight. (c) HPLC chromatogram of ABP hydrolysate (1. Fluctose; 2. Glucose). (d) The hypothesized structure of ABP.

**Fig. S5**

**
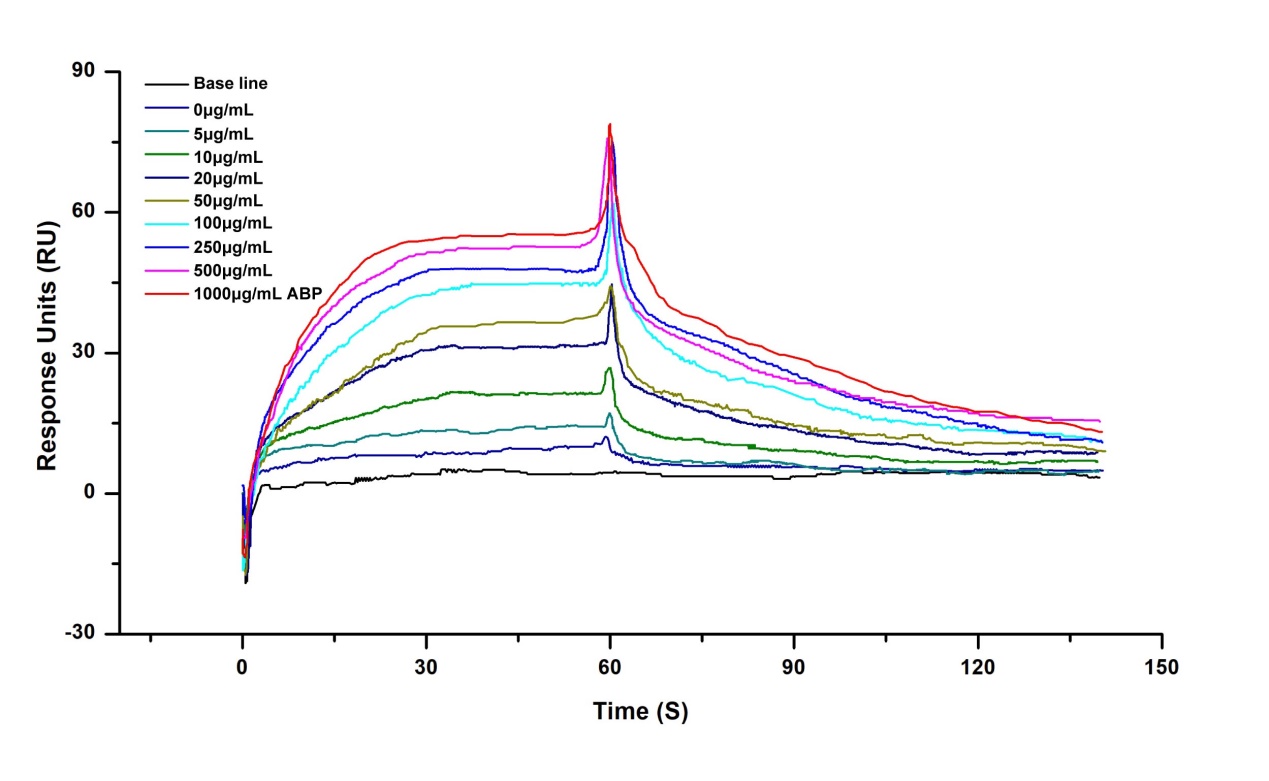
**

**Supplementary Fig. S5.** The concentration-dependent binding of ABP to surface of recombinant EGFR (10 mM) evaluated by the SPR assay.

**Fig. S6**


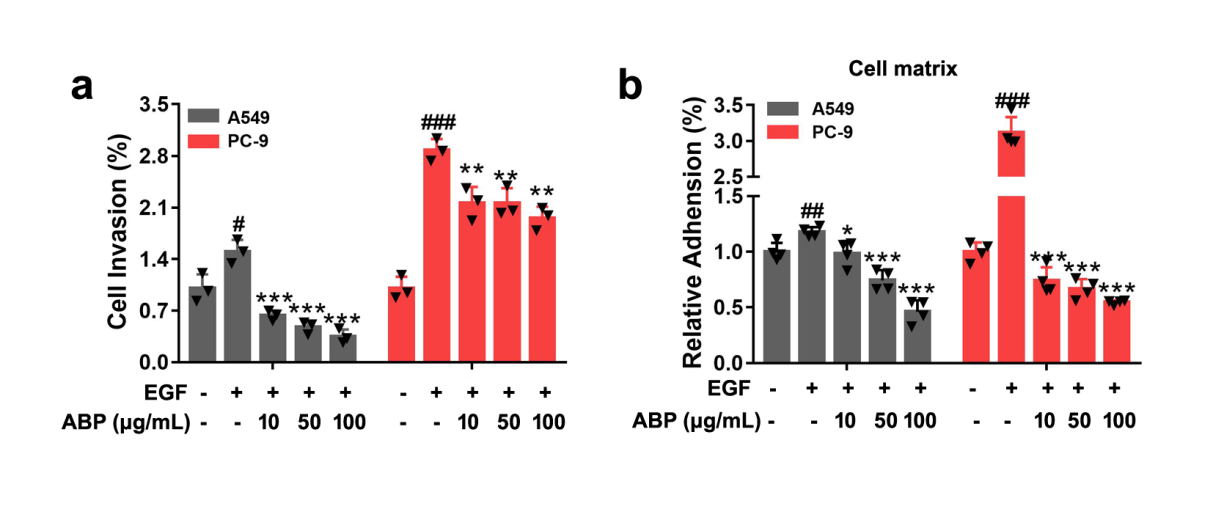


**Supplementary Fig. S6.** (a) After treatment with ABP (10, 50, 100μg/mL) for 24h, cells were exposed to 50ng/mL EGF for 10min. Quantitative analysis the effect of ABP on cell invasion. (b) Quantitative analysis of adhesion of A549 and PC-9 cells to cell-matrix after treatment with ABP in the presence of EGF (20ng/mL). Data represented the mean ± SD, n = 3; *^#^P < 0.05, ^###^P < 0.001* compared to control group; **P < 0.05, **P < 0.01, ***P < 0.001* compared to the EGF treated group.

**Fig. S7**


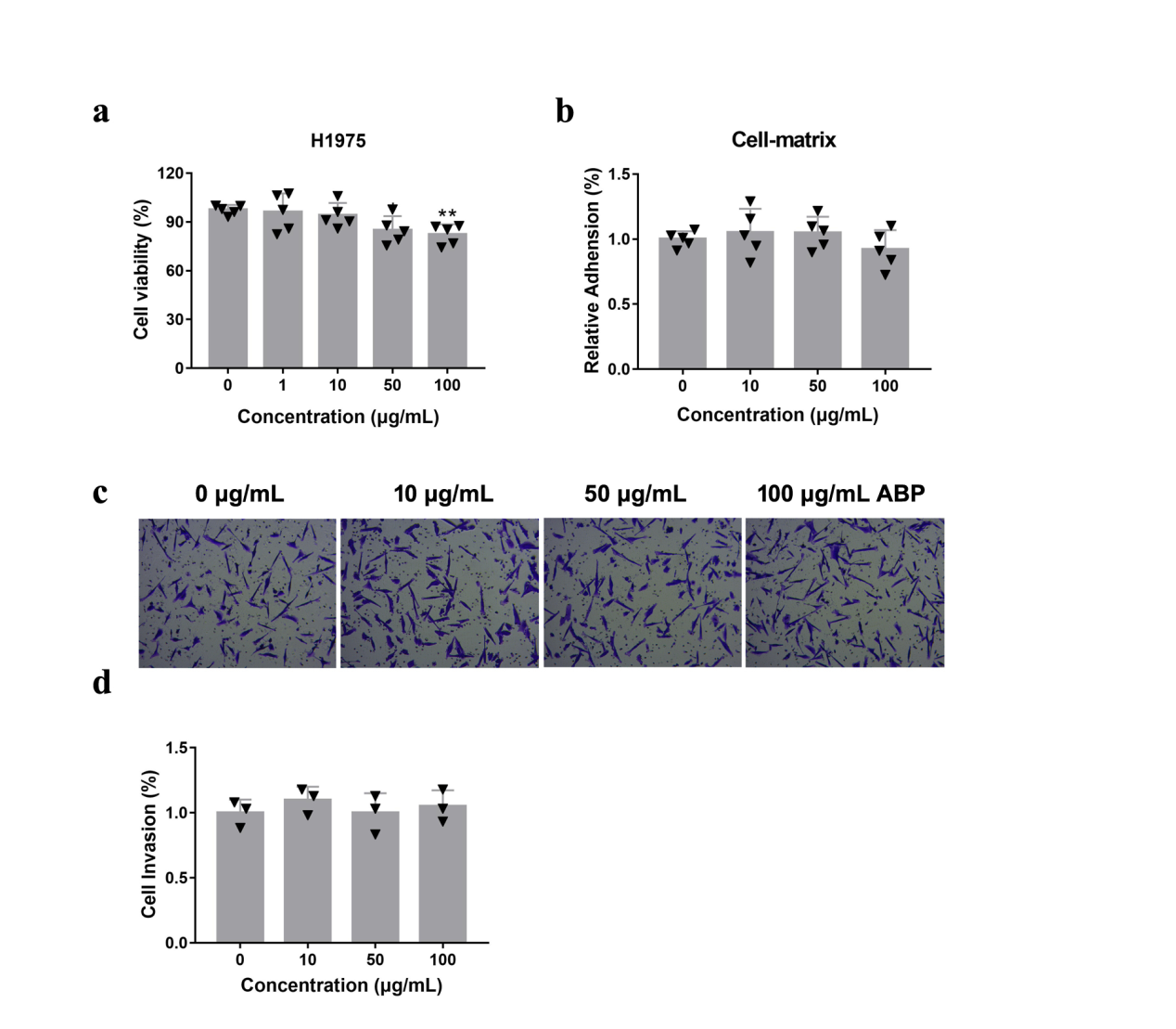


**Supplementary Fig. S7.** **Effects of ABP on adhesion and invasion of H1975 cells.** (a) Effects of ABP on proliferation of H1975 cells. (b) Quantitative analysis of the inhibition of ABP on the adhesion of H1975 cells to cell-matrix. (c) Representative images of the transwell invasion of H1975 cells treated with ABP for 48h. (d) Quantitative analysis of the effect of ABP on H1975 cell invasion. Data represented the mean ± SD, n = 3-5; **P* < 0.05, ***P* < 0.01, compared to the control group.

**Fig. S8**


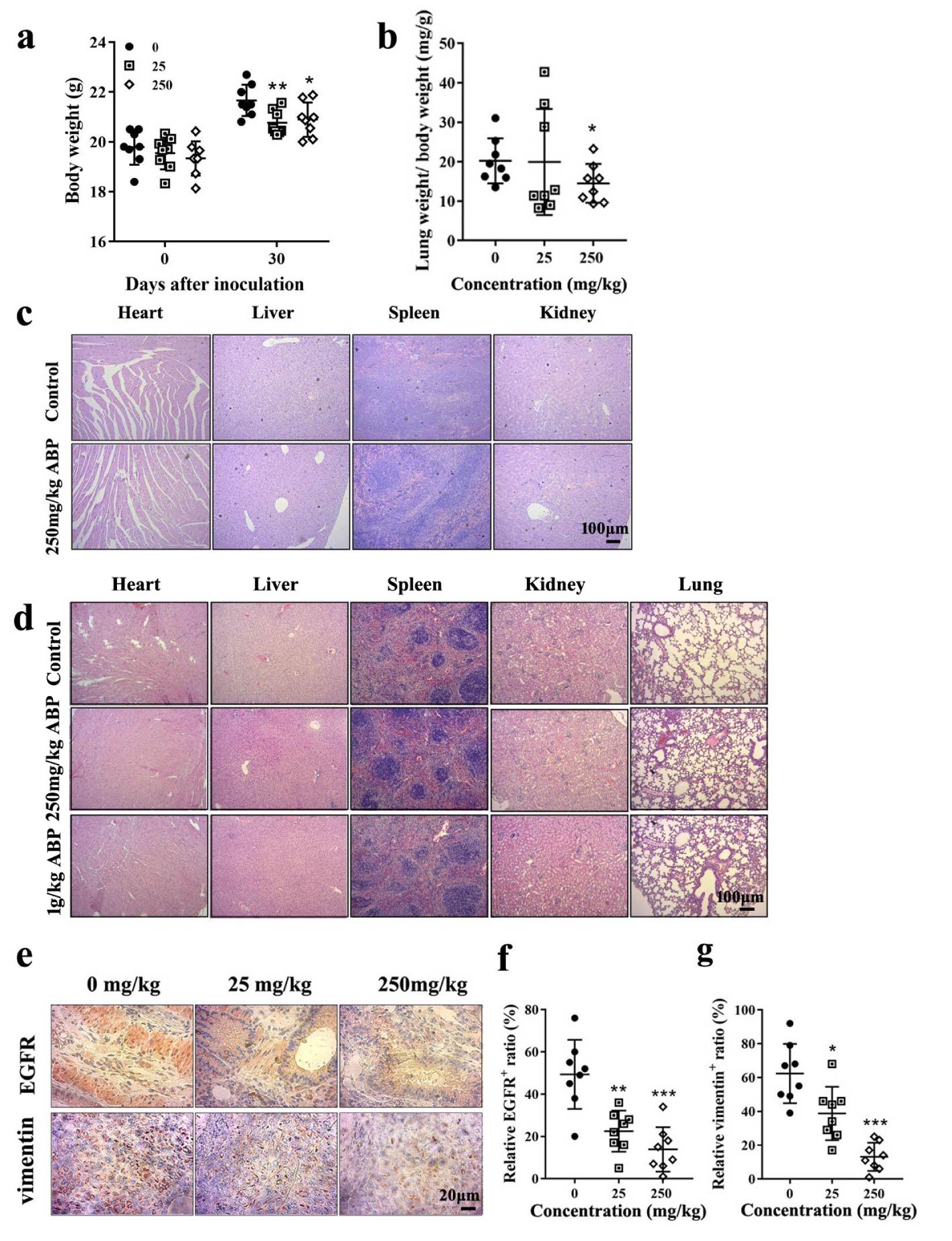


**Supplementary Fig. S8. Effect of ABP on lung cancer metastasis in mice.** After tumors were surgically removed, ABP or PBS was administrated to mice daily for 30 days. (a) Body weight change during the treatment period. (b) The ratio of lung weight to body weight. (c) HE staining of paraffin-embedded sections of the heart, liver, spleen and kidney, amplification ×10. (d) Normal mice were administrated with ABP (0, 250, 1000mg/kg) by gavage for 5 days, and sacrificed for blood and tissue examinations. HE staining of paraffin-embedded sections of the heart, liver, spleen, lung and kidney, amplification ×10. (e) Immunostaining of anti-EGFR and vimentin antibodies on lung sections of control mice and ABP-treated mice, magnification×40. (f-g) Histological quantification of EGFR^+^ and vimentin^+^ cells abundance in lung tissues. Data represented as the mean ± SD, n = 8; **P < 0.05, **P < 0.01, ***P < 0.001* compared to the control group.
